# Hypopituitarism after Orthohantavirus Infection: What is Currently Known?

**DOI:** 10.3390/v11040340

**Published:** 2019-04-10

**Authors:** Soerajja Bhoelan, Thomas Langerak, Danny Noack, Linda van Schinkel, Els van Nood, Eric C.M. van Gorp, Barry Rockx, Marco Goeijenbier

**Affiliations:** 1 Department of Viroscience, Erasmus MC, ‘s-Gravendijkwal 230, 3015 CE Rotterdam, The Netherlands; b.s.Bhoelan@gmail.com (S.B.); Thomas.langerak@erasmusmc.nl (T.L.); d.noack@erasmusmc.nl (D.N.); e.vangorp@erasmusmc.nl (E.C.M.v.G.); b.rockx@erasmusmc.nl (B.R.); 2 Department of Internal Medicine, Erasmus MC, ‘s-Gravendijkwal 230, 3015 CE Rotterdam, The Netherlands; l.gribling-vanschinkel@erasmusmc.nl (L.v.S.); e.vannood@erasmusmc.nl (E.v.N.)

**Keywords:** orthohantavirus, HFRS, hypopituitarism, endocrine disturbances, review

## Abstract

Several case reports have described hypopituitarism following orthohantavirus infection, mostly following Puumala virus. The pathogenesis of this seemingly rare complication of orthohantavirus infection remains unknown. This review explores the possible pathophysiological mechanisms of pituitary damage due to orthohantavirus infection. In only three out of the 28 reported cases, hypopituitarism was detected during active infection. In the remaining cases, detection of pituitary damage was delayed, varying from two months up to thirteen months post-infection. In these cases, hypopituitarism remained undetected during the acute phase of infection or only occurred weeks to months post infection. Both ischemic and hemorrhagic damage of the pituitary gland have been detected in radiographic imaging and post-mortem studies in the studied case reports series. Ischemic damage could be caused by hypotension and/or vasospasms during the acute phase of hemorrhagic fever with renal syndrome (HFRS) while hemorrhage could be caused by thrombocytopenia, thrombopathy, and other known causes of coagulation disorders during orthohantavirus infection. Also, hypophysitis due to the presence of auto-antibodies have been suggested in the literature. In conclusion, a significant number of case reports and series describe hypopituitarism after orthohantavirus infection. In most cases hypopituitarism was diagnosed with a delay and therefore could very well be underreported. Clinicians should be aware of this potential endocrine complication, with substantial morbidity, and if unrecognized, significant mortality.

## 1. Introduction

Hemorrhagic fever with renal syndrome (HFRS) comprises a group of clinically similar rodent-borne infectious diseases caused by orthohantaviruses, including Hantaan (HTNV), Dobrava (DOBV), Seoul (SEOV), Saaremaa (SAAV), and Puumala virus (PUUV) [1]. The clinical course of these infections is classically characterized by a triad of acute renal failure, fever, and hemorrhagic complications. HFRS can be divided into five phases: febrile, hypotensive, oliguric, diuretic, and a convalescent phase [2,3]. Each pathogenic orthohantavirus is carried by a specific rodent and the demographic location of each type of HFRS corresponds to the natural habitat of this rodent species. Generally, HTNV is mainly prevalent in Asia, whereas DOBV is circulating in the Far East and the Balkans. PUUV generally results in a milder disease and is endemic in Scandinavia and Western Europe, Central Europe, and Russia. With a numerous and geographically wide-spread zoonotic reservoir, *Rattus* species, SEOV is the only orthohantavirus prevalent worldwide [3].

Depending on which orthohantavirus species infects a patient, case fatality rates vary from <1% to 15% [2,3]. Besides often observed renal complications, patients may present with extrarenal complications in the respiratory and cardiovascular system while severe hemorrhage may also occur [4]. From the old world hantaviruses, DOBV infections are generally considered to result in the highest percentage of hemorrhagic complications (up to 15–30%), while PUUV is known to cause *nephropathia epidemica* (NE), a syndrome comparable to HFRS with a milder clinical course and mortality rates of 0.1–1% [5].

Several case reports have described hypopituitarism following orthohantavirus infection. The pathogenesis of this complication remains unknown. Already in the 1950s, different post-mortem studies showed pituitary damage, e.g., foci of hemorrhage and necrosis, in 58–72% of patients with hemorrhagic fever, which are now thought to be HFRS cases [6,7]. In 1985, a study by Lim et al. demonstrated pituitary gland atrophy on radiographic imaging in seven out of eleven confirmed HFRS patients [8]. Later, Valtonen et al. described three fatal cases of acute NE in which pituitary gland damage was detected during autopsy [9]. Literature about the global prevalence of pituitary damage following HFRS is scarce, however data suggests that pituitary damage, characterized by endocrinological disturbances including potential life-threatening Addison’s crises, due to orthohantavirus infection is not uncommon. This review aims to explore the possible pathophysiological mechanisms for pituitary damage in orthohantavirus infections in literature and illustrates its clinical relevance.

## 2. Pathophysiology of Hypopituitarism

The pituitary gland produces hormones that control major physiological endocrine systems in the body. The anterior pituitary gland regulates growth via the production of growth hormone, the thyroid function through production of thyroid-stimulation hormone (TSH), the blood pressure (via adrenocorticotropic hormone (ACTH)), and the gonadal function (via luteinizing hormone (LH) and follicle-stimulating hormone (FSH)). In the posterior pituitary cells, the hypothalamic hormones anti-diuretic hormone (ADH) and oxytocin are stored and subsequently released when needed [10].

Hypopituitarism is a condition in which one or more hormones are insufficiently released by the pituitary gland [10,11]. Insufficient ACTH and TSH release can lead to an adrenal crisis or severe hypothyroidism and can be life-threatening [11]. Primary hypopituitarism is caused by pathology of the pituitary gland itself, secondary hypopituitarism is caused by a dysfunctional hypothalamus [11]. Most commonly, primary hypopituitarism is caused by mechanical pressure on (by tumors or edema), or hemorrhage inside, the pituitary gland [10,12,13].

Another known cause of primary hypopituitarism is Sheehan’s syndrome where pituitary necrosis follows postpartum blood loss [14]. During pregnancy, the pituitary enlarges for the purpose of lactation and requires a relatively increased blood supply [15]. Hypovolemia due to postpartum hemorrhaging may lead to insufficient blood supply to the pituitary. Other pathological processes that might lead to pituitary damage in Sheehan’s syndrome include the presence of vasospasm; thrombosis, potentially in the context of disseminated intravascular coagulation (DIC) or peripartum prothrombogenic state; and hypotension [14]. Pituitary failure due to auto-immunity is a relatively rare condition, where inflammation processes are either primarily targeted at the pituitary gland, such as lymphocytic or granulomatous hypophysitis, or occur in systemic auto-immune diseases, such as sarcoidosis or Crohn’s disease, and bacterial or viral infections like HIV and cytomegalovirus infection [16]. In contrast to acute vascular damage to the pituitary gland, an autoimmune mechanism underlying the pituitary gland problems seen post-orthohantavirus infection could explain a more smothering clinical course. For instance, endocrine disturbances caused by auto-antibodies due to orthohantavirus infection has been suggested in a single case report [17].

## 3. Clinical Consequences of Hypopituitarism

Clinical manifestation of hypopituitarism depends on the hormones that are lacking and the rapidity of onset [10]. An overview of clinical features of hypopituitarism is given in Table 1. The general signs in patients with hypopituitarism are nonspecific, such as fatigue and weakness [18]. Dysfunction of the posterior pituitary gland occurs less frequently than dysfunction of the anterior gland. However, posterior pituitary gland dysfunction causing diabetes insipidus due to anti-diuretic hormone (ADH) deficiency does have significant clinical consequences [11,19]. Deficiency of the growth-hormone (GH) usually precedes dysfunction of the gonadotropic axes (LH and FSH) followed by impaired production of TSH and ACTH. Deficiencies of ACTH and TSH are potentially life-threatening, whereas deficiencies of LH, FSH, and GH are associated with chronic morbidity [11,20]. 

Hypopituitarism can be adequately treated using hormone replacement therapy of end-hormones from involved pituitary axes [11,18]. Duration of therapy depends on the reversibility of the damage to the pituitary gland [11]. Specific attention is warranted for glucocorticoid replacement as an increase in dosage is necessary in the case of acute stress and illness, meaning that continuous monitoring and adjustments of hormone replacements are needed in patients with inadequate stress hormone response [10,11].

## 4. Hemorrhagic Complications in Orthohantavirus Infection

It is plausible that the pituitary gland dysfunction observed post orthohantavirus infection is a result of hemorrhagic complications seen in HFRS. Orthohantavirus infections are, like other viral hemorrhagic fevers, associated with a triad of thrombocytopenia, endothelial cell dysfunction, and disseminated intravascular coagulation (DIC) resulting in various hemorrhagic complications [21]. The mechanisms by which these are caused are only partially understood. Clinical observational studies focusing on alterations in primary and secondary hemostasis during orthohantavirus disease showed thrombocytopenia in both HFRS and New World orthohantavirus cardiopulmonary syndrome (HCPS). A decreased plasma activity of coagulation factors II, V, VIII, IX, and X in acute HFRS patients, prolongation of the prothrombin and activated partial thromboplastin time, increased thrombin generation and D-dimer levels, and a decrease in ADAMTS13 activity were observed in PUUV-infected patients [22,23,24,25]. Orthohantaviruses are able to infect endothelial cells in vitro by binding to β3-integrins, a cell surface receptor involved in platelet function and vascular permeability [26,27]. Multiple in vitro studies investigated the role of endothelial cells in orthohantavirus infection. Dendritic cells and macrophages produce pro-inflammatory cytokines during infection, which evoke a changing phenotype of the endothelium towards a pro-adhesive (for immune cells) and pro-coagulant response during inflammation promoting a final procoagulant shift of the endothelium [28]. This causes increased vascular permeability and enhanced recruitment of platelets on the endothelial surface [29].

Furthermore, orthohantaviruses interact with the GPIIa/IIIb receptors on the surface of thrombocytes, which upon binding leads to increased platelet activation [30]. Thereby, a direct binding between PUUV particles and quiescent platelets is possible [31]. In contrast to other hemorrhagic fever viruses, which decrease maturation and stimulate apoptosis of megakaryocytes, orthohantaviruses stimulate HLA expression of megakaryocytes in vitro, making them potential targets for cytotoxic lymphocytes, which on itself potentially decreases the production of platelets [32,33]. Both loss of activated platelets and a decreased platelet production in orthohantavirus infection could result in a decrease in number of circulating platelets and thereby interact with primary hemostasis. In vitro, orthohantavirus infection of endothelial cells directly leads to an increase of tissue factor and subsequent thrombin generation and a decrease of fibrinolysis, resulting in a clear procoagulant state [31] while a suppression of Thrombospondin 1 and an increase of tissue plasminogen activator have also been described [34,35]. In line with in vivo data, an adequate response in secondary hemostasis could very well also be hampered during orthohantavirus infection.

## 5. Clinical Characteristics of Hypopituitarism Due to Orthohantavirus Infection

However, clear bleeding of the pituitary gland does not seem to be the underlying mechanism in all of the reported cases of hypopituitarism after orthohantavirus infection. Direct pituitary damage due to orthohantavirus has been identified and published in 28 patients in 11 case-reports and case-series [9,17,36,37,38,39,40,41,42,43,44]. Thus far, impaired function of the posterior lobe of the pituitary has not been described. An overview and summary of the data retrieved from literature is provided in Table 2.

PUUV infection was reported most frequently (14/28 cases) [9,17,38,39,40,43,44]. While one case report discussed DOBV infection [36], in 14 cases (two originating from Korea [37,41] and twelve originating from Serbia [42,43]), a specific orthohantavirus type was not mentioned since diagnosis was made via serology detecting multiple orthohantaviruses. However, given the geographical distribution of the orthohantavirus serotypes, the two cases originating from Korea might concern HNTV or SEOV, while cases originating from Serbia might concern PUUV, SEOV, or DOBV. It remains unclear whether one of the orthohantaviruses specifically predisposes for this complication. In four cases, the hypopituitarism was detected during active infection [36,37,39,44]. In three of these cases, reasons to suspect an endocrine disturbance during infection were impaired recovery despite improving clinical and biochemical parameters of infection and detection of hypothyroidism during routine thyroid testing [36,37,39]. In the fourth case, the rationale for endocrinal testing during admission was not provided [44]. Radiographic imaging eventually revealed either an atrophic or enlarged pituitary gland with signs of hemorrhage and/or necrosis in all these cases.

In the remaining cases, detection of pituitary damage was delayed varying from a few months to thirteen years after infection [17,38,40,41,42,43]. These patients presented with signs of endocrine disturbances after recovery from orthohantavirus infection. Most frequently reported symptoms were persistent fatigue despite improving renal function or improving hyperlipidemia [43]. Some patients were diagnosed with hypogonadism [38,40,43]. In one case, therapy-resistant ventricular tachycardia led to the discovery of hypopituitarism and subsequent hypothyroidism 13 years after having HFRS, which eventually resolved after hormonal replacement therapy with glucocorticoids and thyroid hormones [41]. Furthermore, one patient suffered initially from hypogonadism and hypothyroidism six months after acute infection. Another six months later this patient developed polydipsia and polyuria due to diabetes insipidus [17].

In twelve cases, pituitary damage was confirmed with either a computerized tomography (CT) or Magnetic resonance imaging (MRI) scan, showing atrophy of the pituitary gland [17,43,44], or even an empty sella [38,43], but an enlarged pituitary with signs of hemorrhage was also noted in some cases [36,39,44]. One patient had a normal aspect of the pituitary gland on the CT scan [40].

All patients experienced acute renal failure. Of these patients sixteen patients required hemodialysis or -filtration [36,37,42,43]. In four cases the clinical course was also complicated by extrarenal manifestations, such as myocardial infarction, pancreatitis, acute respiratory distress syndrome, gastro-intestinal bleeding, DIC, and septic shock [36,40,43].

Lastly, in five cases, pituitary damage was revealed during postmortem examination [9,44]. Both necrotic and hemorrhagic areas were present in the pituitary. In the case of Hautala et al., granulocyte infiltration of the gland was observed [44]. In the cases of Valtonen et al., all patients suffered from diffuse intravascular coagulation [9].

All surviving patients were successfully treated with hormonal replacement. The duration of therapy was not reported in most cases. In two cases, replacement therapy was only required for several months, possibly indicating reversible damage [36,39].

## 6. Pathophysiology of Orthohantavirus Associated Hypopituitarism

The exact pathophysiological mechanism leading to hypopituitarism after an orthohantavirus infection remains to be elucidated; however, there are several theories that can explain this phenomenon. The mechanism in hypopituitarism following orthohantavirus infection was suggested to be analogous to Sheehan’s syndrome after postpartum fluxus, as the acute phase of orthohantavirus infection can be accompanied by hypotension [43,44]. In this theory, necrosis is caused by insufficient vascular supply of the pituitary gland due to hypotension, potentially combined by vasospasm due to septic shock. This is supported by the autopsy findings of Steer et al., which showed 21 out of 36 patients with HFRS who died during the hypotensive phase and all of 28 patients who died in the oliguric phase had pituitary gland damage [6]. A postmortem study by Hullinghorst and Steer from the 1950s did not detect occlusion of the pituitary vasculature of patients who died due to orthohantavirus infection [45]. This finding makes vasospasms resulting from hypotension during or due to orthohantavirus infection a possible cause of pituitary gland necrosis. Proving this theory, however, is difficult as vasospasms cannot be assessed with histopathologic features.

Pituitary damage in orthohantavirus infection could also be caused by a hemorrhage. A study of Stojanovic et al. on hypopituitarism after PUUV infection showed patients with hypopituitarism had significantly lower platelet levels during acute infection compared to patients with normal pituitary function after infection, suggesting an important role of thrombocytopenia in the pathogenesis of hypopituitarism after orthohantavirus infection [42]. Indeed, foci of hemorrhage were found in the pituitary gland after orthohantavirus infection [9]. An association between hypopituitarism and the severity of infection, mainly determined by the duration of the oliguric phase, has been suggested [8]. It seems that thrombocytopenia and uremia give a generalized risk for hemorrhage since an increased uremia level on its own causes thrombocytopathy. However, bleeding complications in hantavirus infection are most likely the result of a more complex interaction between the virus, endothelial cell, and immune system. The question why hemorrhage specifically occurs in the pituitary gland remains unanswered. Jost et al. suggested the existence of hypophysitis in orthohantavirus infection [39]. Additional evidence comes from the study of Hautala et al. in which PUUV orthohantavirus has been isolated from stromal cells and vascular endothelial cells of the pituitary gland [44]. The latter could lead to endothelial damage and dysfunction, making the pituitary gland more prone to vascular damage. However, this result might also suggest direct damage of pituitary cells due to infection. Tarvainen et al., who reported on hypopituitarism six months after acute infection, suggested hypopituitarism to be an auto-immune reaction triggered by PUUV infection as MRI findings compatible with hypophysitis sequela and elevated thyroid peroxidase antibodies were found [17].

One would expect the occurrence and the extent of pituitary damage to be related to the clinical severity of the acute infection. However, a Sheenan-like phenomenon would be the result of persistent hypotension, something hardly observed in PUUV, which usually has a mild and favorable clinical course. Furthermore, a retrospective study of Stojanovic et al., which evaluated the prevalence of hypopituitarism after HFRS, reported a prevalence of 13% in a median of two years after recovery, showing the relatively high prevalence of this complication [42]. The duration of the oliguric phase and occurrence of complications are used to determine the clinical severity of hemorrhagic fever [8]. Severity of pituitary damage is suggested to be related to the stage of the disease. However, Steer et al. demonstrated that 58% of their patients who died during the hypotensive stage had mild pituitary necrosis, whereas 100% of the patients who died during oliguric phase had severe pituitary necrosis [6]. Furthermore, there were also specific cases only showing isolated pituitary necrosis without signs of multi organ failure. On the other hand, thrombopoietin, procoagulant activity, coagulation variables, and platelet indices, e.g., mean platelet volume, are shown not to be predictive of the severity of renal insufficiency and hypotension due to PUUV infection. This further questions the correlation between pituitary damage and the severity of infection [46]. However, thrombocytopenia has shown to be associated with the severity of inflammation, e.g., expressed as a C-reactive protein (CRP), and severity of capillary leakage in acute PUUV infection [47]. Pituitary gland damage (e.g., due to orthohantavirus infection) can be visualized using a CT or MRI. However, one can question whether radiographic imaging observations correspond to the presence and severity of endocrine disturbances. In a clinical study by Lim et al., patients obtained follow-up CT scans during the acute infection and convalescent phase of infection [8]. The severity of pituitary atrophy or necrosis on imaging did not correspond to the level of hormone disturbances in laboratory testing. Thereby, Partanen et al. re-evaluated a cohort of patients four to eight years after acute NE. All patients underwent MRI examinations during acute infection [48]. This study did not reveal any significant hormonal disturbances. Even though abnormal hormonal laboratory testing was found in some patients, these were deviations from reference intervals due to secondary factors, e.g., obesity or use of medication.

## 7. Discussion

**Figure 1:**
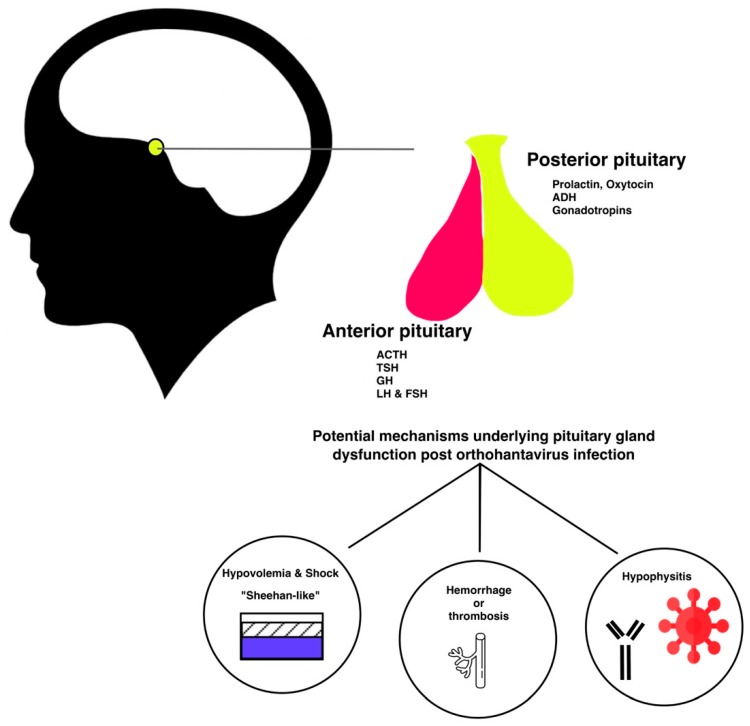
Illustrative summary of potential mechanisms underlying pituitary damage post orthohantavirus infection.

Case reports and series in the past two decades have been associating orthohantavirus infection with hypopituitarism. The fact that patients initially present with nonspecific symptoms or even subclinical disease potentially contributes to delayed diagnosis. Less specific abnormalities in the gonadal and thyroid axis were reported retrospectively in 30 of 54 patients during acute NE, suggesting a significant problem [49]. However, one should keep in mind that a dysbalanced hormonal axis in these 30 patients was not necessary be the result of hypopituitarism. The exact pathophysiological mechanisms leading to hypopituitarism during or following orthohantavirus infection remains unknown. While also preexisting endocrinological pathology could be detected once a patient presents to the hospital with HFRS as described in [50]. We have summarized potential underlying mechanisms in Figure 1. Autopsy cases and radiographic imaging have confirmed both necrotic and hemorrhagic damage of the pituitary gland [6,7,8]. However, long term follow-up did not always confirm the persistency of hormonal abnormalities [49]. The group of cases reporting late-onset hypopituitarism did not take other confounders into account. Orthohantavirus can lead to ischemic, necrotic, and hemorrhagic damage of the pituitary gland. The precise pathophysiological mechanism leading to this damage is to date unclear. However, several mechanisms have been explored in literature and are summarized in Figure 1. Ischemic damage could be caused by hypotension and/or vasospasms, and hemorrhagic damage could be caused by thrombocytopenia and thrombopathy. Also, the presence of hypophysitis and auto-antibodies post orthohantavirus infection have been suggested. Since all data thus far relies on observational studies with low numbers of patients, it is impossible to make strong recommendations. However, there seems to be a significant association between pituitary gland dysfunction and hantavirus infection. Several questions need to be answered. For instance, in vitro or ex vivo infection with hantavirus of pituitary gland cells could confirm the potential tropism of orthohantavirus for the pituitary gland. Meanwhile, for discriminating between vascular or auto-immune mechanisms, an animal model could be of interest. In conclusion, hypopituitarism after orthohantavirus infection is frequently diagnosed with a delay and is possibly under-reported or -detected. Clinicians should be more aware of this complication as endocrine disturbances can severely affect a patient’s wellbeing.

## Figures and Tables

**Table 1:** Clinical features of hypopituitarism [10,11,19,20].

Hormone Deficiency	Signs and Symptoms
Growth hormone (GH)	Increased body fat; reduced muscle mass and strength; reduced stamina and psychological problems, e.g., depression or concentration loss; dyslipidemia; atherosclerosis
Luteinizing and follicle-stimulating hormone (LH and FSH)	Sub- or infertility, loss of libido Men: impotence; testicle atrophy; loss of facial, body, and pubic hair; reduced muscle mass; osteoporosis Women: a- or oligomenorrhea, dyspareunia, osteoporosis
Thyroid-stimulation hormone (TSH)	Cold intolerance, weight gain, fatigue, hair loss, constipation, hoarse voice
Adrenocorticotropic hormone (ACTH)	(Orthostatic) hypotension, hypoglycemia, fatigue, muscle weakness
Prolactin (PL)	Postpartum failure of lactation
Antidiuretic hormone (ADH)	Polydipsia and polyuria

**Table 2:** Summary of the 28 cases of post orthohantivurs panhypopituitarism reported in literature. NE-IFAT: nephropathia epidemica indirect immunofluorescence assay test.

Serotype	Country	No.	Diagnostics	Endocrine Disturbances	Time to Detection	Duration	Outcome	Ref
**Puumala**	Finland	1	NE-IFAT titre	Panhypopituarism	5 years	NR	Survived	Forslund et al., 1992 [38]
**Puumala**	Sweden	1	Specific Puumala IgM and IgG	Panhypopituitarism	6 months	NR	Survived	Settergen et al., 1992 [40]
**NR**	Korea	1	Serologic antibody testing	Panhypopituitarism	Day 20 ^a^	NR	Survived	Suh et al., 1994 [37]
**Puumala**	Finland	4	NR	NR	-	-	Deceased	Valtonen et al., 1995 ^b^ [9]
**NR**	Korea	1	NR	Panhypopituitarism	13 years	NR	Survived	Kim et al., 2001 [41]
**Puumala ^b^** **Puumala** **Puumala**	Finland Finland Finland	1 1 1	Serologic antibody testing Serologic antibody testing Serologic antibody testing	NR Panhypopituitarism Panhypopituitarism	- 5 months Day 7 ^a^	- NR NR	Deceased Survived Survived	Hautala et al., 2002 [44]
**NR** **Puumala** **Puumala**	Serbia Serbia Serbia	1 1 1	Serologic antibody testing Serologic antibody testing Serologic antibody testing	Panhypopituitarism Panhypopituitarism ACTH, FSH, LH and GH	1.5 years 2 years 2 years	NR NR NR	Survived Survived Survived	Pekic et al., 2005 [42]
**NR** **NR** **NR** **NR**	Serbia Serbia Serbia Serbia	3 2 1 5	Indirect immunofluorescent assay Indirect immunofluorescent assay Indirect immunofluorescent assay Indirect immunofluorescent assay	GH FSH, LH ACTH ≥4 axes	>6 months >6 months >6 months 0.5–11 yrs	NR NR NR NR	Survived Survived Survived Survived	Stojanovic et al., 2008 [42]
**Puumala**	Austria	1	Specific Puumala IgM	Panhypopituitarism	Acute ^a^	5 months	Survived	Jost et al., 2009 [39]
**Dobrova**	Turkey	1	Serologic antibody testing	Panhypopituitarism	Day 19 ^a^	16 months	Survived	Sariguzel et al., 2010 [36]
**Puumala**	Finland	1	Specific Puumala IgM and IgG	TSH, FSH, LH, ADH	6 months	Ongoing	Survived	Tarvainen et al., 2016 [17]

^a^ Hormonal disturbances detected during acute infection; ^b^ Post-mortem study revealing pituitary hemorrhage. NR: not reported.
